# Primary constrained and hinged total knee arthroplasty: 2- and 5-year revision risk compared with unconstrained total knee arthroplasty: a report on 401 cases from the Norwegian Arthroplasty Register 1994–2017

**DOI:** 10.1080/17453674.2019.1627638

**Published:** 2019-06-18

**Authors:** Mona Badawy, Anne Marie Fenstad, Ove Furnes

**Affiliations:** a Coastal Hospital in Hagavik, Department of Orthopaedic Surgery, Haukeland University Hospital;; b Norwegian Arthroplasty Register, Department of Orthopaedic Surgery, Haukeland University Hospital;; c Department of Orthopaedic Surgery, Haukeland University Hospital;; d Department of Clinical Medicine, Faculty of Medicine, University of Bergen, 5021 Bergen, Norway

## Abstract

Background and purpose — The number of primary, highly constrained knee arthroplasty implants has increased with a theoretically increased risk of early failure. Therefore we analyzed the risk of all revision following total knee arthroplasty (TKA) in patients receiving a hinged or condylar constrained knee (CCK) compared with a conventional unconstrained TKA.

Patients and methods — The analyses included 401 primary highly constrained or hinged implants from 1994 to 2017. Kaplan–Meier survival curves were used to evaluate time to first revision with a maximum follow-up of 20 years. Cox regression was used to calculate hazard ratio (HR) comparing condylar constrained knee (CCK), hinged, and unconstrained TKA.

Results — Kaplan–Meier estimated prosthesis survival after 2 years was 94.8% (95% CI 91.4–98.2) and 93.5% after 5 years for the primary CCK and 91.0% (CI 86.6–95.4) after 2 years and 85.5% after 5 years for the primary hinged TKA. Adjusted for sex, age groups, diagnosis, time period, previous surgery, and surgery time HR was 1.4 (CI 0.8–2.3) for the CCK and 2.4 (CI 1.6–3.7) for the hinged implants. The most common cause of revision in hinged implants was infection: 14 of 22 revisions. When excluding infection as revision cause, there were no differences in survival between the implant types. Estimated survival excluding infection revisions at 5 years was 96% for unconstrained, CCK, and hinged primary TKA implants.

Interpretation — Primary rotating hinge total knee arthroplasty had a higher risk of revision compared with conventional TKA after 2 and 5 years’ follow-up. Infection was the most common cause of revision. When excluding infection revisions from the survival analysis, hinged and CCK implants had similar performance to unconstrained TKA.

Selective use of varus and valgus constrained or rotating-hinge implants in primary total knee arthroplasty is necessary in complex cases with severe ligament laxity, bone loss or deformity. The current awareness of planning for the right type of implant constraint has increased its use beyond salvage revision indication (National Joint Registry [NJR] 2017, Norwegian Arthroplasty Register [NAR] 2018).

Instability is a major cause of revision in conventional total knee arthroplasty (Parratte and Pagnano 2008, Dyrhovden et al. 2017), particularly in younger patients (Victor 2017). Secondary osteoarthritis due to previous trauma and surgery is a major cause for knee replacement in the younger population. In these cases, constrained implants might be necessary to achieve a stable knee. The need for aggressive ligament releases in patients with major deformities or contractures may also require implants such as constrained and rotating-hinge designs (Westrich et al. 2000, Yang et al. 2012, Ghosh et al. 2016).

Total knee arthroplasty surgery is predicted to increase worldwide and concurrently there is an increase in the use of primary constrained implants because of the awareness of the importance of obtaining primary stability. There are studies from single institutions (Petrou et al. 2004, Gehrke et al. 2014, Farid et al. 2015, Cottino et al. 2017), but the rates of failure leading to revision surgery have previously only been evaluated in 1 registry study to our knowledge (Baker et al. 2014). Due to the limited use of these implants and the heterogeneity of the studies, it is difficult to obtain certain conclusions regarding the long-term results. We analyzed the survival and revision causes in a large cohort of these complex primary total knee implants to provide objective evidence.

## Patients and method

### Data collection

**Figure 1.**
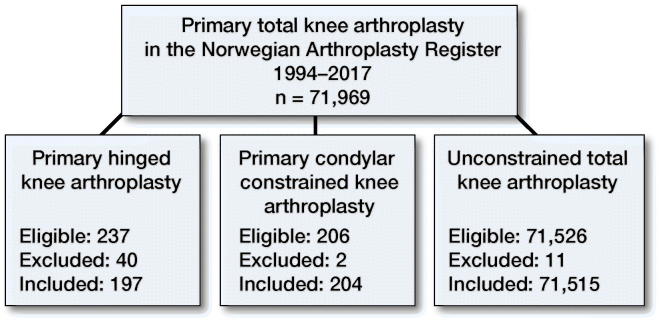
401 cases of constrained or hinged implants were included from the Norwegian Arthroplasty Register from 1994 to 2017. 53 cases were excluded due to oncological indication for surgery. digits are number of implants.

The Norwegian Arthroplasty Register has collected data on knee arthroplasty surgery since 1994 (Furnes et al. 2002). Type of procedure such as primary or revision, unconstrained or constrained/hinged, the use of stems and augments in addition to implant brand and indication for surgery is recorded. Using this information, condylar constrained knee implants and hinged knee replacements used for primary total knee arthroplasty submitted to the NAR from January 1994 until December 31, 2017 were identified. We used the stabilization of the polyethylene as definition of constraint. The implant library of the Norwegian Arthroplasty Register has listed the polyethylene inserts as minimally stabilized (CR, dished), posterior stabilized (S), rotating platform, constrained condylar (CCK), and hinged (only rotating hinged had been used) verified by catalogue numbers of the implants. Minimally stabilized and posterior stabilized rotating platform and fixed bearing implants were defined as unconstrained TKA. Patient and surgery information were available for analyses in 401 cases of constrained or hinged implants (Figure 1).

Revision of the TKA was defined in the registry as exchange, complete or partial removal, or addition of implant component(s), and information on the indications for revision was obtained as well. The status of each knee replacement was assessed as revised, unrevised, or death of the patient. Information on death was retrieved from the National Population Register. Revision was linked to the primary procedure using the unique national identification number of the patient. Implant survival at 10 years was determined using revision for any reason as primary endpoint. Secondary endpoint was revisions excluding infections, analyzing aseptic reasons for revision separately. Since the percentage of hinged and CCK implants increased from 2005 and the majority were used in this time period, we did a sensitivity analysis including only ASA 1 and ASA 2 patients from 2005 to 2017 using the same adjustments (minus ASA classification) in the Cox model as mentioned in the statistical analyses.

### Statistics

The Kaplan–Meier method was used for estimation of survival probabilities for the implants, with 95% confidence intervals (CI) with a maximum follow-up after 10 years. Cox regression analysis was used to calculate hazard ratios (HR) to estimate the survival rates adjusted for sex, diagnosis, age groups, time period, surgery time, previous surgery, perioperative complications, and ASA classification. These are presented with CIs relative to the conventional unconstrained TKA. Proportional hazard assumptions of the Cox regression model were assessed by tests and inspection of Schoenfeld residuals (Ranstam and Robertsson 2010). P-values less than 0.05 were considered statistically significant and all tests were 2-sided.

### Ethics, funding, and potential conflicts of interest

The Norwegian Arthroplasty Register has permission from the Norwegian Data Inspectorate to collect patient data based on written consent from the patient (ref 24.1.2017: 16/01622-3/CDG). The authors received no specific funding for this work. No conflicts of interest were declared.

## Results

**Figure 2.**
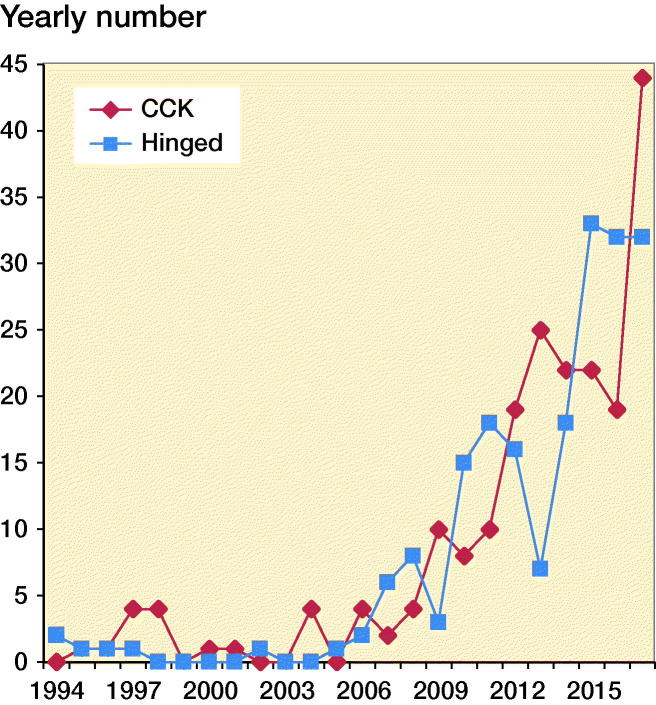
Increasing usage of primary CCK and hinged TKA in the from 1994 to 2017 in the Norwegian Arthroplasty Register.

From 1994 to 2017 primary hinged TKA was used in 197 primary cases and 22 cases (11%) were revised. Figure 2 demonstrates the increased use of primary hinged TKA and CCK since 1994. From 2005 there was an increased use until today, with 32 hinged and 42 CCK primary TKA in 2017. Regarding patient and procedure characteristics there were fewer male patients receiving constrained implants than conventional TKA (28% vs. 36%) (Table 1). Hinged TKA and CCK were more commonly used in young patients (< 50 years) and in the oldest patients (> 80 years) (Table 1).

**Table 1.** Demographic data (n = 71,916) by TKA implant type from 1994 to 2017. Values are frequency (%) unless otherwise specified

	Hinged	CCK	Unconstrained^a^	p-value^b^
Primary procedures	197	204	71,515	
Revisions	22 (11)	14 (7)	3,565 (5)	< 0.001
Male sex	55 (28)	58 (28)	25,423 (36)	0.008
Age, years mean/median range	67/70	67/68	69/70	< 0.001
	22–90	25–95	16–101	
Age groups (%)				< 0.001
< 50	14	11	4	
50–60	15	17	14	
60–70	23	28	32	
70–80	32	27	38	
> 80	16	17	12	
Diagnosis^c^				< 0.001
Primary OA	64 (33)	109 (53)	59,023 (83)	
Inflammatory arthritis	15 (8)	15 (7)	4,347 (6)	
Post-fracture arthritis	28 (14)	22 (11)	1,926 (3)	
Post ligament injury	37 (19)	38 (19)	5,161 (7)	
Post infection	9 (5)	5 (2.5)	232 (0.3)	
Instability	13 (6)	1 (0.5)	49 (0.1)	
Neuro orthop. sequelae	10 (5)	2 (1)	25 (0.1)	
Other	20 (10)	12 (6)	627 (0.5)	
Time period				< 0.001
1994–2007	15 (8)	21 (10)	25,942 (36)	
2008–2017	182 (92)	183 (90)	45,573 (64)	
Surgery time^c^ (minutes)				< 0.001
median (IQR)	150 (55)	145 (55)	90 (35)	
range	85–420	80–360	31–654	
ASA classification^d^				< 0.001
ASA 1	16 (9)	14 (8)	7,313 (14)	
ASA 2	94 (51)	107 (58)	35,066 (66)	
ASA 3+	74 (40)	64 (33)	10,587 (20)	
Previous surgery				< 0.001
None	122 (62)	127 (62)	50,788 (71)	
Fracture	34 (17)	29 (14)	1,402 (2)	
Ligament	20 (10)	19 (9)	9,005 (13)	
Osteotomy	4 (2)	11 (5)	2,393 (39	
Other	17 (9)	18 (9)	7,927 (11)	
Perioperative complications^c^	7 (3.6)	17 (8.5)	1,331 (1.9)	< 0.001
Patellar component^e^	34 (17)	40 (20)	5,235 (7)	< 0.001
Stems femur/tibia, n^f^	155/150	167/164	527/4,290	
Augments femur/tibia, n^g^	16/18	24/16	785/763	

a Unconstrained TKA were procedures with cruciate retaining or posterior stabilized, mobile or fixed bearing TKA.

b 2-sided t-test for continuous variables. Chi-square test for categorical variables. Independent sample median test = non-parametric.

c Diagnosis missing n = 126, surgery time missing n = 1,813, and perioperative complications missing n = 1,275.

d From 2005, n = 53,335

e TKA with patellar component

f 18% of information regarding use of stems was missing for hinged implants, 15% missing for CCK, and 42% missing for unconstrained TKA in the registry data.

g 58% of information regarding use of augments was missing for hinged implants, 56% missing for CCK, and 43% missing for unconstrained TKA in the registry data.

Primary TKA with tumor indication were removed from the data material (n = 40 hinged, n = 2 CCK, n = 11 unconstrained).

The Kaplan–Meier 2- and 5-year survival free of all revisions for primary hinged TKA was 91.0%. The CCK 2- and 5-year survival was 94.8%, comparable to 5-year survival of unconstrained TKA, which was 95.3%. Hinged primary TKA had a shorter follow-up with the last revision at 6 years (Table 2). The 10-year survival for primary CCK declined to 78.9% compared with conventional TKA, which had a 10-year survival of 93.7%.

**Table 2a.** Kaplan–Meier survival^a^ free of all cause revision at 2, 5, and 10 years postoperatively

	No. of patients	No. of revisions (%)	No. of deaths (%)	No. at risk	K–M 2-yea survival (CI)	No. of risk	K–M 5-year survival (CI)	No. at risk	K–M 10-year survival (CI)
Unconstrained	71,515	3,565 (5)	15,669 (22)	57,585	97 (97–98)	39,936	95 (95–96)	17,379	94 (94–94)
CCK	204	14 (7)	31 (15)	128	95 (91–98)	57	94 (89–98)	8	79 (62–96)
Hinged	197	22 (11)	19 (10)	115	91 (87–95)	58	86 (79–92)	8	84 (77–91)

a Kaplan–Meier survival (%) with 95% CI in parentheses for all-cause reoperation for the entire follow-up period.

**Table 2b.** Unadjusted and adjusted hazard ratio^a^

	Unadjusted HR	Adjusted HR
Unconstrained	1 (ref.)	1 (ref.)
CCK	2.0 (1.2–3.3)	1.4 (0.8–2.3)
Hinged	3.3 (2.2–4.9)	2.4 (1.6–3.7)

a Hazard ratio is shown unadjusted and adjusted for sex, age groups, diagnosis, time period, surgery time, previous surgery to the knee, perioperative complications, and ASA classification (registered since 2005 in the register).

The adjusted hazard ratio (HR) for primary hinged TKA as compared with conventional TKA was 2.4. HR for primary CCK versus conventional TKA was 1.4 (Table 2).

Primary osteoarthritis was the diagnosis leading to surgery for 83% of patients in unconstrained TKA compared with only 33% receiving hinged TKA. Post-fracture osteoarthritis and post-ligament injury osteoarthritis were dominating causes for surgery in the hinged (33%) and CCK (30%) TKA groups. Post-infection osteoarthritis was the preoperative diagnosis in 5% of hinged TKA cases versus 0.3% in conventional TKA (Table 1). The anterior cruciate ligament was reported to be deficient preoperatively in 56% of hinged TKA cases, 47% in CCK cases, and 19% in unconstrained TKA. The posterior cruciate ligament was deficient preoperatively in 32% of hinged cases, 24% in CCK cases, and 2% in unconstrained TKA cases (NAR 2018). Patients with no previous surgery to the knee were 62% in the hinged and CCK group versus 71% in the conventional TKA group. 2% in the conventional TKA group were previously surgically treated for fracture near the joint, whereas 14% and 17% had previous fracture treatment in the hinged and CCK group respectively. ASA 3+ patients were registered in 40% of the hinged knee patient group whereas only 20% were ASA 3+ in the conventional TKA group. Median surgery time in the hinged and CCK group was 150 and 145 minutes respectively, versus 90 minutes for conventional TKA.

7 (4%) and 17 (9%) cases of perioperative complications were reported in the hinged and CCK group respectively, versus 1,331 (1.9%) in conventional TKA (Table 1). The reported types of perioperative complications are demonstrated in Table 3 (see Supplementary data). There was a high percentage of fractures and tendon ruptures perioperatively as compared with unconstrained TKA.

There were no differences in HR comparing female with male patients (reference) (HR = 1.0 (CI 0.1–1.0). Young patient (< 50 years) had a higher risk of revision HR = 2.2 (CI 1.9–2.5), and older patients (> 80 years) had a lower risk of revision HR = 0.6 (CI 0.5–0.6) using age 60–70 as reference. Post-infection osteoarthritis had a higher risk of revision, HR = 1.8 (CI 1.3–2.6) (not shown in table).

In primary hinged TKA, infection was the dominant reason for revision. 16 cases were revised due to deep infection. 5 revisions of CCK were due to deep infection (Tables 4 and 5, see Supplementary data).

**Figure 3.**
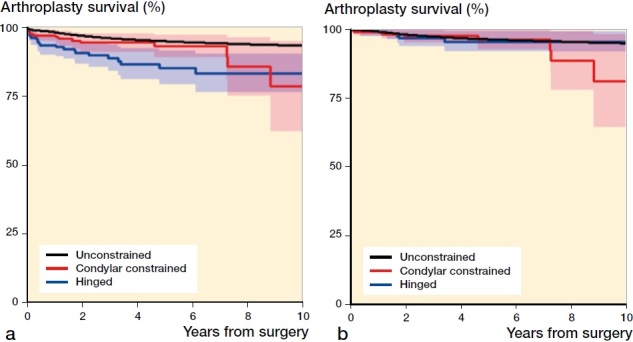
Kaplan–Meier survival curve with revision for any reason (a) and excluding infection revision (b) from 1994 to 2017 for TKA by implant type; blue = primary hinged knee replacement, red = primary condylar constrained knee replacement, black = unconstrained total knee replacement.

Figure 3a shows the inferior implant survival of primary hinged TKA as compared with unconstrained TKA with revision for any reason as endpoint. Figure 3b demonstrates all revisions excluding infection revisions, showing the similarity in survival comparing the 3 implant types when the most common cause of revision was removed from the data. Kaplan–Meier estimated survival for aseptic revisions at 2 years was 98.1% (CI 97.9–98.3) for unconstrained TKA, 97.6% (CI 95.2–100) for CCK, and 96.7% (CI 93.7–99.7) for hinged TKA. K–M 5-year survival for aseptic revisions was 96% for all 3 groups for TKA.

**Table:** Number at risk (Figure 3)

Year	0	1	2	3	4	5	6	7	8	9	10
Hinged	197	154	116	86	67	59	47	30	19	15	9
CCK	204	153	128	108	82	57	37	27	19	10	8
Unconstrained	71,515	64,503	57,585	51,179	45,282	39,937	34,792	30,132	25,584	21,166	17,380

In the sensitivity analysis including the latest time period from 2005 to 2017 including only ASA 1 and ASA 2 patients, we found similar results for all revisions as in the complete analysis. HR for CCK as compared with unconstrained TKA as reference was 1.5 (CI 0.7–3.1), whereas for hinged implants HR was 2.3 (CI 1.2–4.2).

## Discussion

The principal findings in this study were higher reoperation and revision rates in patients undergoing complex primary total knee replacements as compared with conventional total knee arthroplasties. Most commonly, revisions were caused by infection.

The adjusted relative risk of all-cause revision at 10 years was > 2 times higher in patients receiving a hinged implant compared with patients receiving an unconstrained conventional implant at the time of the index surgical procedure.

The condylar constrained knee implants had good survivorship, comparable to unconstrained TKA. The mid-term survival of primary hinges in this cohort at 5 years was statistically significantly inferior to unconstrained implants. However, a separate analysis of aseptic revisions showed similar survival after 5 years for all implant types.

The assumption that revision implants used in the primary setting would perform as well as standard implants was not found in this study, probably due to patient selection and characteristics. The differences in results comparing unadjusted and adjusted analyses indicates that the higher risk of revision is attributable to factors other than implant design.

Patients undergoing primary hinged and constrained implants typically had secondary osteoarthritis caused by previous fractures, ligament injuries, and infections; only one-third had primary osteoarthritis as indication for surgery. There were more young patients (< 50 years) and more old patients (> 80 years) as well as more ASA 3+ patients in the hinged and constrained cohort compared with patients receiving routine primary knee arthroplasty. Surgery time was also prolonged in these more complex knee implants, which can be explained by both the complexity of the implant itself and the abnormality of the preoperative deformity and instability.

The increased risk of infection could be explained by the high amount of ASA 3+ patients, combined with prolonged surgery time with more soft tissue exposure and trauma (Badawy et al. 2017). We found that post-infection osteoarthritis had a higher risk of revision. This is supported by the proceedings of international consensus on orthopedic infections (Aalirezaie et al. 2019). A registry study from Finland also found increased risk of infection in patients with hinged or constrained implants (Jämsen et al. 2009). The results of our study should, however, not discourage surgeons from using constrained implants when considered necessary to achieve a stable knee. The high risk of infection should lead to prevention measures both pre- and perioperatively, optimizing the patient preoperatively and performing atraumatic surgery to avoid hematoma formation due to poor soft tissue handling and avoiding perioperative complications such as fractures and ligament injuries. We found a higher risk of perioperative complications in the hinged and constrained knee implant groups.

Our study supports the findings of previous series where the most common reason for revision in complex primary knee arthroplasty was infection (Petrou 2004, Yang et al. 2012, Baker et al. 2014, Cholewinski et al. 2015, Martin et al. 2016, Cottino 2017, Siqueira et al. 2017).

A study from the NJR (Baker et al. 2014) found that the implant survival for hinged knee replacements was comparable to conventional knee replacements, in contrast to our study, whereas others found the risk of revision to be higher in constrained primary implants (Moussa et al. 2017). The NJR study reported a high number of older patients (mean age 72) with primary osteoarthritis (70%) with hinged TKA, contrary to our study with only 33% reported primary OA. Some studies report low rates of aseptic loosening in rotating hinge TKA, but fail to offer conclusions regarding cases of infection revisions (Westrich et al. 2000, Cottino et al. 2017). Comparable to our study, Cottino et al. (2017) and Rai et al. (2018)found a high number of patients with intraoperative complications such as fractures and ligament injuries.

First-generation rotating hinge total knee designs were associated with a high failure rate (Rand et al. 1987, Barrack 2001). More recent designs have improved the rotating hinge mechanism, the patellofemoral tracking, metaphyseal sleeves, and cones, and improved articulation between the mobile-bearing element and the tibial component (Barrack 2001, Deehan et al. 2008, Smith et al. 2013). There seems to be a high satisfaction rate among primary constrained and hinged knee arthroplasty patients. PROMs data in the NJR study by Baker et al. 2014 demonstrated improvements in function and general health outcomes following surgery.

CCK has potential disadvantages due to increased constraint and is thought to have a higher risk of aseptic loosening due to the tight fit of the insert post in the femoral component. Rai et al. (2018) found similar implant survival (95%) to our study and with a high complication rate for CCK. CCK used by Sabatini et al. (2017) showed good functional results in their series of 28 patients. Cholewinski et al. 2015 also demonstrated a high risk of infection revisions in CCK and, similar to our study, revision for reasons other than infection was close to values of unconstrained implants. They discussed the possible decreasing level of constraint over time due to polyethylene creep at the tibial post, thus mechanical long-term complications such as loosening did not occur.

There are a number of limitations to interpretation of our data. The number of hinged and constrained implants accounted for a small number of the total amount of primary knee arthroplasty in Norway. Thus, the generalizability of the results is limited. We did not have PROMs data, so revision for any reason was the endpoint for our results. Even in the NJR study (Baker et al. 2014), where PROMs exists as a source of information in the national register, the information was available only for a small proportion of patients. There is also a heterogeneity in the cohort, since many patients had previous and varying surgical interventions. Even if we had made adjustments for this in the analysis, there could be a selection bias. The infection revisions are mostly soft tissue debridement with exchange of polyethylene insert, indicating early infection and revision. However, we have no information on whether there actually was bacterial growth in specimens after surgery, verifying the diagnosis. This should not, however, influence the comparison between implant types (Gundtoft et al. 2015). A higher threshold to revise such complex implants could lead to falsely high survival rates due to the complexity of revision. However, regarding infection revisions, there could be a lower threshold to do soft tissue revisions due to wound drainage at an earlier stage than with unconstrained TKA.

In summary, there has been an increased use of primary hinged and condylar constrained total knee arthroplasty in the last decade. These implants should always be considered in complex cases to achieve a stable knee. Rates of septic failure are higher than for conventional total knee arthroplasty, probably caused by a higher number of patients with comorbidities, and also due to mechanical and soft tissue challenges in addition to the size and complexity of the implant, increasing the surgical duration and risk of haematoma formation in these complex cases. When excluding infection revisions from the survival curve, hinged and CCK implants showed similar performance to unconstrained TKA. The patients’ general risk of infection should be optimized, as should the surgeons’ skills regarding correct indication and use of these more complicated implants to ensure results comparable to conventional TKA.

### Supplementary data

Tables 3–5 are available as supplementary data in the online version of this article, http://dx.doi.org/10.1080/17453674.2019.1627638

The study was conceived by MB, AMF, and ONF, MB wrote the initial draft, AMF performed the analyses, all authors contributed to the interpretation of the data and to revision of the manuscript.

Acta thanks Esa Jämsen and Maziar Mohaddes for help with peer review of this study.

## Supplementary Material

Supplemental Material
