# Supplementary material for: Primary constrained and hinged total knee arthroplasty: 2- and 5-year revision risk compared with unconstrained total knee arthroplasty: a report on 401 cases from the Norwegian Arthroplasty Register 1994–2017
Source: Acta Orthop. 2019 Jun 18;90(5):467–72. doi: 10.1080/17453674.2019.1627638 (PMC6746286; doi:10.1080/17453674.2019.1627638)
Supplement: Supplemental Material [file IORT_A_1627638_SM6754.pdf]

Supplementary data

Table 3. Perioperative complicationsa by implant type

Type of complication	Hinged	CCK	Unconstrained
Heart related	0	0	24
Bleeding	0	0	25
Anesthesiological problems	0	2	39
Patient related	0	2	144
Tourniquet	1	0	49
Technical problems/instruments	2	2	269
Fractures	4	3	210
Patellar tendon rupture	0	2	161
MCL rupture/damage	0	4	86
Instability	0	2	14
UKA converted to TKA	0	0	16
Other	0	0	40
Missing	0	0	254
Total, n (%)	7 (3.6%)	17 (8.5%)	1,331 (1.9%)

[a] Perioperative complications is a no/yes question in the registry knee replacement form. If the surgeon ticks yes, there is a free-hand field to be filled out on which type of complication.

Table 4. Revision causes and type of revision by implant type (number of revisions)

Factor	Hinged n = 22	CCK n = 14	Unconstrained n = 3.565
Revision causes			
Deep infection	16	5	802
Loose proximal and distal	0	0	142
Loose proximal	1	0	102
Loose distal	0	1	525
Fracture (near prosthesis)	2	0	151
Dislocation/loose patella	1	0	95
Dislocation (not patella)	0	0	49
Instability/malalignment	0	1	722
Polyethylene wear	1	4	103
Pain only	0	2	559
Other	1	1	289
Missing	0	0	26
Type of revision			
Exchange of polyethylene	10	8	902
Exchange of all components	3	1	1,121
Exchange of distal component	1	0	415
Exchange of proximal component	2	0	105
Implantation of patellar component	1	2	501
Exchange of patellar component	0	1	27
Soft tissue debridement	1	1	34
Removal of implant part(s)	3	1	305
Other	1	0	155

Table 5. Implant brands by implant type from 1994 to 2017

Hinged	n	CCK	n	Unconstrained TKA	n
NexGen Rotating Hinge	154	NexGen LCCK	135	Profix	14,709
RT-Plus Modular	16	Triathlon TS	19	NexGen	14,337
Legion Hinge Knee	9	AGC dual	14	LCS complete	13,035
KOTZ	6	Scorpio TS	9	AGC	4,866
Modular Rotating Hinge	4	LCS Complete VVS	7	LCS	4,818
S-ROM Rotating Hinge	4	PFC-Sigma TC3	4	PFC-Sigma	3,375
Link Endo Model-M Hinged	2	Dual articular 2000	4	Genesis I	3,293
Link Endo-Model Total Rotation	1	Vanguard TM	4	Legion	3,095
Mutars	1	Profix constrained	3	Triathlon	2,920
		Vanguard 360 revision	2	Duracon	2,751
		Legion constrained	2	Other	4,316
		Duracon TS	1		
Total	197		204		71,515
